# A case–control study of occupational contact levels in the childhood leukaemia cluster at Seascale, Cumbria, UK

**DOI:** 10.1136/bmjopen-2015-008432

**Published:** 2015-08-04

**Authors:** Leo J Kinlen

**Affiliations:** The Nuffield Department of Population Health, University of Oxford, Oxford, UK

**Keywords:** EPIDEMIOLOGY

## Abstract

**Objectives:**

To investigate adult occupational contact levels and risk of childhood leukaemia and non-Hodgkin's lymphoma (LNHL) in Seascale, an association found in other situations of rural population mixing (PM).

**Design:**

Matched case–control study.

**Setting:**

Seascale, Cumbria, UK.

**Participants:**

For each case of LNHL recorded in patients under age 25 years during 1950–2006, up to 20 matched controls were chosen and parental occupational details obtained; an exception was a single working young adult, whose own occupation (and that of controls) was used.

**Primary outcome measures:**

Contact levels of occupations were categorised as: low/medium (reference group), high or very high contact levels, as in previous studies, with provision for certain unusual occupations. In particular, specialist policemen responsible for security and access at the nearby Sellafield nuclear complex were allocated to the highest contact category, and those Sellafield employees who worked in controlled areas to the middle (high) category. Since of possible bias, unusual contact aspects noted in the main research and development (R&D) building were reserved for a supplementary analysis. ORs were calculated for the occupational contact levels.

**Results:**

Compared to the reference group, the social class adjusted ORs for the high and very high contact categories were 8.18 (95% CI 0.95 to 70.33) and 14.90 (1.20 to 184.90), respectively, with a significant trend across the categories (p value=0.024). In the supplementary analysis with R&D workers moved to the very high contact category, the OR for the latter became 29.68 (2.12 to 415.79), and the p value for trend, 0.011.

**Conclusions:**

The Seascale LNHL excess was most marked among those young people linked with high occupational contact levels; it is therefore not an exception to the pattern of family infection shown by other PM-related excesses. The findings have implications for the choice of controls in certain types of virus study.

**Strengths and limitations of this study**

- This case–control study focuses on the notable and well-publicised excess of leukaemia and non-Hodgkin's lymphoma (LNHL) among young people who have lived in the remote rural village of Seascale, Cumbria, UK, which has experienced high levels of population mixing (PM).
- In other situations of rural (but not urban) PM, excesses of LNHL among young people have been found to be greatest in those linked to high levels of adult occupational contacts, and this pattern is confirmed by the present study.
- The number of cases of LNHL in Seascale is limited and the measures of occupational contacts restricted to the data available, but the results of this study are consistent with previous findings of studies of other PM-associated excesses using a similar approach.
- This study increases the evidence for LNHL in young people being a rare response to a common (but currently unidentified) infection, and encourages efforts to search for and identify the responsible infectious agent(s).
- Owing to the evidence of family infection, family members should not be selected as controls in leukaemia virus studies, and urban controls are also inappropriate because of the evidence of widespread urban immunity.

## Introduction

No cluster of childhood leukaemia (CL) has made a more striking impact than that discovered by a television team in 1983 at Seascale near the remote Sellafield nuclear complex in Cumbria, North-west England, and broadcast in prime viewing time. Government action was prompt, with an independent official inquiry quickly established, which confirmed an approximately tenfold excess of CL. Initially, attention focused on the role of radiation, but doses to children in the village from Sellafield radioactive discharges were less than those received from natural background and less than 1% of the level required to explain the excess.^1^ In its report, the inquiry recommended that a case–control study be carried out in west Cumbria, and also the establishment of the Committee on Medical Aspects of Radiation in the Environment (COMARE), an independent expert committee to advise the UK Government. The increased incidence was found to extend to young adults (aged 15–24 years), and to non-Hodgkin's lymphoma (NHL), with which CL has been confused, particularly in the past, prompting the recommendation by COMARE that in relevant studies both disorders (leukaemia and NHL, LNHL) should be considered.^2^

In 1990, the case–control study aforementioned^3^ reported an association between LNHL and paternal preconceptional irradiation (PPI) at Sellafield, which was suggested as the cause of what is often termed ‘the Seascale cluster’. However, PPI could not explain the significant excess in children who had come into the village after birth elsewhere,^4^ nor was the cluster compatible with the distribution of PPI doses in west Cumbria;^5^ more generally, no support for a PPI relation with CL has been found.^6^ ^7^

A quite different hypothesis also emerged—that the excess was caused by infection promoted by rural population mixing (PM) of which isolated Seascale was an extreme example, since it received large influxes to work at nearby Sellafield. The PM hypothesis proposed that if, as in many viral infections, CL is a rare response to a widespread infection (here unidentified), marked influxes to rural areas (especially if isolated) would promote new contacts between infected and susceptible individuals (the latter more prevalent in rural areas), with consequent increases in the rare response CL.^8^ The houses built in Seascale for Sellafield workers were assigned mainly to administrators and scientists, resulting in it becoming the rural parish with (by far) the highest proportion of social class I residents in the country.^8^ ^9^ The hygiene standards of this group would, as in paralytic poliomyelitis and infectious mononucleosis, tend to promote a high prevalence of susceptible individuals. Other major rural influxes in Britain over the past 70 years have been studied, connected with wartime evacuation, new towns, industrial projects and military camps, and each has revealed a significant increase of CL or LNHL; studies in other countries have also supported the relation.^10^ The important role of adults in infective transmissions in rural PM associated with CL excesses was apparent when the influxes included no children, as in the North Sea oil industry,^11^ and the wartime military occupation of Orkney and Shetland.^12^

Adult transmission was also indicated by the fact that the excesses of CL were highest in the children of parents with ‘high contact’ occupations,^13^ reflecting family infection; similar observations have been made in infections such as cytomegalovirus infection and poliomyelitis.^13^ ^14^ The only occupational details so far reported from Seascale, however, have concerned radiation workers and PPI,^3^ which might suggest that the excess there represents an exception to the association reported in other PM-related excesses. To close this gap, a study of occupational contacts has been carried out, the second case–control study of the Seascale cases since the first was reported 25 years ago.^3^

## Methods

### Cases

The cases investigated were the 13 cases of LNHL under 25 years of age associated with Seascale, while under 25 years of age at diagnosis; they were recorded in the period 1950–2006, though in fact all were diagnosed before 1992.^7^ ^15^ ^16^ These cases were ascertained from a national series of leukaemia death certificates assembled by the Medical Research Council and from local clinicians^4^; all have previously been listed in COMARE reports,^7^ ^16^ though (as here) anonymously. They comprise 11 cases resident in Seascale at diagnosis, together with 2 cases diagnosed within a few months of leaving the village. The fact that these two cases are responsible for a significantly raised incidence of LNHL among ex-residents, and that no additional cases were diagnosed in the period 1950–1991, suggests that they should be treated as cases associated with residence in Seascale.^4^ (Not included is the case of NHL in a young woman who was born in Seascale, mentioned by COMARE,^7^ since the diagnosis was made (after 1991) many years after leaving the village.)

### Controls

Up to 20 age-matched and sex-matched potential controls were selected for each case, a number of controls considered reasonable for achieving adequate statistical power, given the probable attrition. For those born in Seascale who were of (or near) school age, controls with birth dates (before and after) close to that of the case were selected from relevant school registers. For younger cases born in Seascale, controls were chosen by the then Office of Population Censuses and Surveys (OPCS) from the birth registers for the area. For the cases born outside Seascale, all of whom arrived during their school years, class lists were again used to choose controls having, as in these cases, ‘late’ admission dates to the school in question. For these children, birth certificates were obtained to allow matching as appropriate for cases born in Cumbria but outside Seascale, or England outside Cumbria. Considerable movements occurred both into and out of Seascale,^9^ and controls were only retained if electoral rolls indicated that the parents were Seascale residents for the whole period from birth (or arrival in Seascale) to diagnosis of the relevant case; replacements were not sought for ineligible controls. No personal contact was made with any case or control.

Parental occupational details were sought for all cases and controls, except for the only working young adult in the cluster (aged 23 years at diagnosis), where it was judged that the individual's own occupational contacts should take precedence over those of parents; for this reason, the individual's own occupation was used, as were those of associated controls. The father of one case was a teacher, but in the other 11 cases, the fathers worked at Sellafield, as did the fathers of most controls, and as did the oldest case; for these individuals, occupational details were supplied in 1994 by British Nuclear Fuels plc (BNFL, the then owners of the Sellafield site) with appropriate agreement from the workforce. Brief details were also provided of maternal occupation, which were supplemented by lists of teachers previously published by a local historian,^17^ who also provided other information (N Ramsden, personal communications 2010, 2014). The Sellafield details included work locations within the site (with relevant calendar periods), and an indication of whether the individual was based in an ‘active area’ (ie, a work area with potential for radioactive contamination) and therefore routinely used a change room (see (B) below). Occasionally, work details were missing, when the occupation was recorded in the analyses as ‘unknown’ and allocated to the reference category. For the relatively few individuals who did not work at Sellafield, the paternal occupation was taken from the child's birth certificate.

### Occupational contact categories

There is clearly no objective method of measuring an individual's level of contacts in the past, but the contrasting contact levels in different occupations offer possible, if inexact, surrogates. Each prediagnosis occupation of cases and controls was therefore categorised in terms of its estimated contact level into one of three broad categories, along the lines followed previously:^13^ ^18^ ^19^ (1) *low, medium and unknown*; (2) *high* and (3) *very high* occupational contact levels. The high contact category included salesmen and those providing services to many different people, while the highest (‘very high’) category included teachers, transport-related workers and those in the construction industry; other occupations were classified as low or medium. Apart from the young adult case (and associated controls) whose *own* occupation was used in the analysis, the contact level of either the father or mother was taken, whichever was the higher.

Whereas in previous contact studies, only the occupational title was known, more detail was available here. Provision was made for two occupational groups not covered by the above classification:

The UK Atomic Energy Authority (UKAEA) Constabulary was responsible for security on the Sellafield site, controlling access at the gates, through which thousands of workers passed twice daily, besides patrolling the site and having special search powers. In view of the differences from civil police, UKAEA policemen were placed in the very high contact category because of their necessarily high level of contacts with Sellafield workers.

- Work in the ‘active areas’ of Sellafield potentially involves exposure to radioactive materials**,** so that entry to, and exit from, these areas is controlled via a few barriered change rooms, to avoid radioactive contamination of areas outside the ‘active areas’. Workers based in ‘active areas’ were regarded as having (at least) a high contact level, because of their additional regular contacts within (and near) the limited number of change rooms; they were allocated to the high (middle) contact category.

Enquiries about any places on the Sellafield site other than change rooms, which would have promoted interpersonal contacts, brought to notice aspects of work in the main research and development (R&D) building. A large part of this building (‘B229’) consisted of windowless laboratories; it was also unusual in having a separate technical library and a small refreshment facility to save its workers having to visit the main site library and canteen, aspects that would promote further close contact among the staff, besides a degree of separation from other workers. The radiation work there was specialist in nature, and mainly carried out by men of professional grade, many of whom lived in Seascale; no other building was considered to have as many Seascale residents as the R&D building. The effect of high social class on the level of susceptibles may be seen as tending to make a high contact level in relative terms as ‘very high’.

Since the above details of R&D workers touched on the main aspects of the PM hypothesis, they called for some notice in this study. It was learnt, however, that there had been talk in the neighbourhood about the fathers of certain affected cases having worked in the R&D building. Consequently, although all the above widely known details of this building were elicited by direct questions, there was no means of excluding the possibility that local knowledge of these fathers had, in some way, influenced the attention given to this building. For this reason, the allocation of R&D workers to the very high contact category was reserved for a subsidiary analysis.

In the early 1990s, before work on occupations in PM situations had begun, details were collected from local residents on various aspects of life in Seascale. Repeatedly, mention was made of the intense social activity and crowding in the heavily patronised Seascale Social Club, virtually the only such venue in the vicinity in the earlier years of Sellafield nuclear operations. In the light of this, though only in the supplementary analysis, the manager of the bar of this club, who figured among the parents of an early case, was moved from the high to the very high contact category, as were a few controls with similar or related occupations (though in less frequented places).

### Statistics

The matched case–control data set was analysed by conditional logistic regression, using Stata statistical software.^20^ The contact analyses were adjusted for social class as derived from a General Register Office publication, giving the corresponding social class for each occupation.^21^

### Ethics

A presentation was made to workforce and management representatives at Sellafield before permission was given for the study, the recognised procedure for this type of occupational study in the early 1990s.

## Results

Table 1 shows details of the total of 13 cases of LNHL associated with residence in Seascale that were recorded at ages 0–24 years in 1950–2006, though in fact all were diagnosed before 1992; they have been tabulated previously.^4^ ^7^ ^15^ ^16^ Most cases born in Seascale had lived there for 1–5 years before diagnosis, and this was similar for those who were born elsewhere. The number of controls per case varied from 19 to 8, mainly because of losses occasioned by controls moving from Seascale before the diagnosis of the corresponding case. Occupational details were obtained for all but 13 controls, who were (conservatively) included in the low/medium contact category.

**Table 1:** Seascale-associated cases of LNHL recorded below age 25 years in the period 1950–2006: diagnostic and other details

Case Number	Diagnosis: calendar year (age in years)	Sex	Born in Seascale^4^	COMARE reference*	Other diagnosis details
1	SLL: 1954 (3)	F	Yes	C	A few months after leaving Seascale
2	ALL: 1955 (7)	F	No	D	
3	NHL: 1955 (2)	F	Yes	E	
4	AML: 1960 (2)	M	Yes	G	
5	ALL: 1968 (11)	M	No	H	
6	ALL: 1968 (4)	M	Yes	I	
7	ALL: 1971 (2)	F	Yes	J	
8	CML: 1978 (19)	M	Yes	L	A few months after leaving Seascale
9	ALL: 1979 (5)	F	Yes	M	
10	NHL: 1983 (9)	M	No	N	
11	NHL: 1984 (1)	F	Yes	O	In ‘THORP years’†
12	NHL: 1988 (23)	F	No	Q	In ‘THORP years’†
13	ALL: 1991‡(16)	M	No	S	In ‘THORP years’†

*Table 2.1.^7^

†THORP construction period (1983–1992), post-‘Black Report’.

‡Peak year for numbers of construction workers on the Sellafield site.

ALL, acute lymphatic leukaemia; AML, acute myeloid leukaemia; CML, chronic myeloid leukaemia; COMARE, Committee on Medical Aspects of Radiation in the Environment; F, female; LNHL, leukaemia and non-Hodgkin's lymphoma; M, male; SLL, subacute lymphatic leukaemia; THORP, Thermal Oxide Reprocessing Plant.

Compared to the reference category, comprising low, medium and unknown contact levels, the high and very high contact groups showed significantly raised OR of 10.78 (95% CI 1.18 to 98.43) and 21.26 (2.26 to 199.78), respectively, with a significant trend (p value=0.003) across the categories (table 2). After adjusting for social class, the ORs were somewhat attenuated: for the middle category 8.18 (0.95 to 70.33), and for the very high category 14.90 (1.20 to 184.90); the p value for trend was 0.024.

**Table 2:** Main analysis: occupational contact (OC) categories and leukaemia and non-Hodgkin's lymphoma risk

OC category	Cases	Controls	OR (CI)	OR adjusted for social class (CI)
Low/medium*	1	84	1.00 (reference)	1.00 (reference)
High	7	73	10.78 (1.18 to 8.43)	8.18 (0.95 to 70.33)
Very high	5	26	21.26 (2.26 to 9.78)	14.90 (1.20 to 184.90)
Total	13	183	p value for trend=0.003	p value for trend=0.024

*Including 13 controls with uncertain or unknown occupations.

Numbers, OR and 95% CIs.

In the supplementary analysis, with R&D and bar workers moved into the very high contact group, the ORs for the high and very high categories became 5.25 (0.43 to 63.93) and 21.24 (2.45 to 183.82), respectively; the p value for trend was 0.002 (table 3). After social class adjustment, these became, respectively, 2.96 (0.24 to 37.22) and 29.68 (2.12 to 415.79), and the p value for trend, 0.011.

**Table 3:** Supplementary analysis: occupational contact (OC) categories and leukaemia and non-Hodgkin's lymphoma risk

OC category	Cases	Controls	OR (CI)	OR adjusted for social class (CI)
Low/medium*	1	84	1.00 (reference)	1.00 (reference)
High	2	42	5.25 (0.43 to 63.93)	2.96 (0.24 to 37.22)
Very high	10	57	21.24 (2.45 to 183.82)	29.68 (2.12 to 415.79)
Total	13	183	p value for trend=0.002	p value for trend=0.011

*Including 13 controls with uncertain or unknown occupations.

Numbers, OR and 95% CIs.

## Discussion

This study finds evidence of an occupational contact effect in the Seascale excess of LNHL as reported in other PM-related excesses,^13^ and in very rural areas of Scotland and Sweden.^18^ ^19^ As before,^13^ ^18^ ^19^ teachers and the construction industry (in the highest contact category) were represented (in three cases). The close and prolonged contacts of teachers with children and (their often subclinical) infections need no elaboration, while in the construction industry, aspects of hygiene and the regular changes in workmates and job location, often away from home, make for a high level of new and close contacts. They have figured in epidemics of various infections,^22^ ^23^ as well as in the epidemiology of cervical cancer, in which the high prevalence of away-from-home (including construction) workers among spouses^24^ has wider relevance than specifically sexual contacts.^13^ A construction industry worker was based at the hub of the construction at Sellafield (in 1983–1992) of the Thermal Oxide Reprocessing Plant (THORP), perhaps Britain's largest rural project, when more than 50 000 passes were issued (including replacements).^25^ The construction of THORP, coinciding with a very high incidence of LNHL, occurred *after* the discovery of the cluster, the statistical significance of which was therefore unaffected by the earlier (*post hoc*) observations. Another two cases involved the children of UKAEA policemen, a group with close contacts on the site with (the up to 9000) Sellafield employees, as well as with construction workers. A notable feature of table 1, and also of a recent study,^15^ is the absence of any case of LNHL in Seascale in the period 1992–2006, when the number of construction workers had declined dramatically with the completion of THORP; this is entirely consistent with the PM hypothesis.

The Sellafield R&D building can be seen as a microcosm of aspects of Seascale highlighted in the PM hypothesis, namely, the high social class of its (incomer) occupants, the promotion of close contacts among them, and a degree of separation from the main workforce with which there were inevitably contacts in the main canteen and at the site gates. The association of paralytic poliomyelitis in adults with higher socioeconomic status indicates how high standards of hygiene can prevent early viral exposure and allow persistence of susceptible status into adult life.^26^ This has more than theoretical relevance here since, in a national study of servicemen in the 1950s, paralytic poliomyelitis was the only infective disorder to show a similar relation with rural PM as CL.^27^ A special examination of R&D workers was reserved for a supplementary analysis, partly because of the possibility of bias (see Methods section), but even without this, the main analysis shows that Seascale is not an exception to the pattern of high occupational contacts in PM-associated excesses.^13^ Essentially, this is only a particular aspect of the (largely subclinical) miniepidemics produced by rural PM, of which a meta-analysis of 17 studies has found a significant excess of CL or LNHL, 13 of them individually significant (4 outside Britain).^10^ With other evidence, such as that from an independent study of the effect of PM in Seascale,^28^ the PM hypothesis has been considered as established.^29^

The present study has a somewhat complicated history: an occupational contact effect was not part of the PM hypothesis as first defined,^8^ but came to light in the course of its testing,^13^ and after most of the present data had been assembled; its collection was initially part of efforts to elucidate the then recently reported association between LNHL risk and PPI.^3^ The COMARE Fourth Report in 1996^16^ restricted its attention to the high incidence of LNHL in Seascale, instead of also examining an area *around Sellafield*, the reason for the original interest and discovery; this led to invalid comparisons with the findings of PM studies. In fact, the excess of LNHL in the area around Sellafield (including Seascale) was similar in magnitude to that found in areas around other large rural industrial projects, which included particular parishes with rates as high as those in Seascale.^25^ ^30^ The COMARE report also stated (among other errors^30^) that appreciable PM occurred in Seascale during the war years, and saw the absence of an associated case of CL as evidence against the PM hypothesis.^16^ As a consequence, substantial effort was diverted to studying wartime PM throughout west Cumbria, which showed that supposed wartime PM in Seascale was based on incorrect information.^31^ A return to the present data was prompted by a recent COMARE enquiry about past occupation-related investigations in the Sellafield area, as part of the Committee's current programme of work.

The Seascale excess involved incoming children as well as those born in the village, the excess in each being significant.^4^ High levels of PPI had featured so strongly among LNHL cases in Seascale^3^ mainly because of the exclusion of children born outside west Cumbria (without, or with little, PPI); the association is most readily explained by chance. In contrast, high contact occupations figure both among those born in, and outside, Seascale, as well as in the most recent three (posthypothesis) cases associated with THORP construction. Although the excess of LNHL among children born outside Seascale is often associated with the case against PPI, this has wider implications, since it indicates that whatever causes CL can operate *after* birth. This was also noted among children born before, as well as during, the wartime PM in Orkney and Shetland.^12^ The parental occupational contact effect in PM studies of CL indicates a high prevalence of *family* infection, with which it is virtually synonymous. This has implications for certain virus studies, namely the unsuitability of family members as controls.

The striking resistance of urban areas to the effects of PM points to widespread immunity in such areas^10^ ^32^ and has a counterpart in the absence of occupational contact effects in (most) studies in the general population, since they are inevitably dominated by urban places.^13^ ^33^ ^34^ An unusual positive effect reported in a recent such study may reflect the power of its exceptional size (15 785 cases).^35^ These urban–rural differences also parallel US experience in the First World War: army camps composed of city-bred recruits had far fewer epidemics than those camps drawing from sparsely settled states.^36^ Both sets of observations point to widespread immunity in urban areas, which means that in viral studies, urban areas are also unsuitable as a source of controls: they should be selected from remote rural areas where children are unlikely to have encountered the relevant infection. No prospective virus study has been reported from an excess associated with PM, and, perhaps surprisingly, none was recommended by COMARE. It is also unfortunate that one of the few recent searches for a novel virus in CL, by the method of redirectional analysis, used family members of metropolitan cases as controls.^37^

## Conclusion

The striking cluster of LNHL among young people in Seascale has been previously linked with the highly unusual PM experienced by this remote rural village. The present study shows that it is not an exception to the association of high occupational contact levels with risk found in other examples of marked rural PM. It also underlines the inappropriateness of choosing controls in certain virus studies from family members or from urban areas. No CL cluster has been as productive of aetiological insights as that at Seascale: that marked PM can produce CL excesses in rural areas, to which urban areas are relatively resistant (as would be predicted of an infective-based disorder); that this cause can operate after birth; and that there is a role for adult transmission and family infection.
